# The impacts of anxiety and depression on outcomes in orthopaedic trauma surgery: a narrative review

**DOI:** 10.1097/MS9.0000000000001307

**Published:** 2023-10-02

**Authors:** Jonathan Weinerman, Arianna Vazquez, Nicolette Schurhoff, Connor Shatz, Brandon Goldenberg, David Constantinescu, Giselle M. Hernandez

**Affiliations:** a Department of Education, The University of Miami Leonard M. Miller School of Medicine; b Department of Orthopaedic Surgery, University of Miami Hospital, Miami, Florida, USA

**Keywords:** amputation traumatic, anxiety, depression, opioid-related disorders, orthopaedic procedures, stress disorders post-traumatic

## Abstract

**Introduction:**

The impact of anxiety and depression on outcomes in orthopaedic trauma surgery is a topic of growing research interest.

**Patients and methods:**

Orthopaedic trauma patients often experience high rates of psychiatric disorders, with anxiety and depression being the most prevalent. Mental health disorders have been shown to increase the risk of negative surgical outcomes and morbidity. This narrative review seeks to summarize the current literature surrounding the impacts of anxiety and depression on orthopaedic trauma surgery outcomes.

**Discussion:**

There is a bidirectional relationship between chronic pain and mental health disorders, involving overlapping brain regions and neurotransmitter pathways. Anxiety and depression have been identified as predictors of negative surgical outcomes in orthopaedic trauma patients. Screening tools like the Patient Health Questionnaire-9 (PHQ-9), Generalized Anxiety Disorder Screener-7 (GAD-7), and Medical Outcomes Study 36-item Short Form (SF-36) can assess mental health status and help tailor interventions. Psychological distress, chronic pain, and traumatic limb amputation are factors that contribute to adverse mental health outcomes in orthopaedic trauma patients. Opioid use for pain management is common in orthopaedic surgery, but it can worsen symptoms of depression and lead to dependency. Non-opioid pain management strategies may improve postoperative outcomes by reducing the impact of opioid-exacerbated depression.

**Conclusion:**

Mental health interventions, both preoperative and postoperative, are crucial in optimizing surgical outcomes and improving patient quality of life. Multidisciplinary approaches that address both physical and mental health are recommended for orthopaedic trauma patients. Further research is needed to develop effective interventions for improving mental health outcomes in this patient population.

## Introduction

Highlights

- Up to 45% of orthopaedic trauma patients may be affected by some type of psychiatric disorder, with anxiety and depression being the most common.
- Mental health disorders increase morbidity and mortality in surgical procedures, and anxiety and depression worsen surgical outcomes in orthopaedic trauma patients.
- Chronic pain and mental health disorders influence each other in a bidirectional manner, with overlapping brain regions and neurotransmitter pathways.
- Psychological distress affects one in five orthopaedic trauma patients, and chronic pain is a frequent side effect of orthopaedic trauma surgeries.
- Opioid use in orthopaedic trauma patients can lead to tolerance, dependence, and an increased risk of chronic opioid use, especially in patients with depression.

There is a growing body of research on the impact of mental health disorders on musculoskeletal health, particularly concerning anxiety and depression^1^. Orthopaedic trauma patients, in particular, have reported rates of psychiatric disorders as high as 45%^2^. Anxiety, characterized by excessive worry and apprehension, is the most prevalent mental health concern in the United States, affecting ~19% of US adults and 7% of US children according to the Diagnostic and Statistical Manual of Mental Disorders, Fifth Edition (DSM-5.)^3^ Similarly, depression, a mood disorder characterized by persistent feelings of sadness and hopelessness, is on the rise, with nearly 20% of adolescents and young adults experiencing depressive symptoms and 1 in 10 US adults experiencing feelings of depression^3,4^.

Mental health disorders have been shown to be common adverse outcomes of surgical procedures, and they increase the risk of morbidity and mortality^5^. Negative mental health outcomes are particularly prevalent among the millions of individuals suffering from orthopaedic injuries each year^6^. Anxiety and depression have been identified as predictors of negative surgical outcomes following orthopaedic injuries, and their impact on surgical outcomes has been studied in several orthopaedic subspecialties^7^. However, there is a lack of research on the effects of anxiety and depression in the context of orthopaedic trauma surgery.

The purpose of this review is to provide context for the pathophysiology of physical and mental health and highlight the need for mental health interventions for orthopaedic trauma patients. By exploring the existing literature for the impact of anxiety and depression on orthopaedic trauma surgeries, we hope to provide a comprehensive overview of the current state of knowledge in this area. Specifically, we will examine the relationships between anxiety, depression, and surgical outcomes in orthopaedic trauma patients, with a focus on preoperative and postoperative mental health interventions that can be acted upon by orthopaedic surgeons and other healthcare professionals who care for patients with orthopaedic trauma injuries.

## Methods

This review is a collation of research findings seeking to summarize the existing literature surrounding the impacts of anxiety and depression on orthopaedic trauma surgery outcomes. A comprehensive search of peer-reviewed journals and books was completed using keywords and MeSH terms to search in databases such as PubMed, Google Scholar, Science Direct, and MEDLINE. The following MeSH terms were used: amputation traumatic, anxiety, depression, opioid-related disorders, orthopaedic procedures, and stress disorders post-traumatic. Generated studies were screened for relevance and reviewed for inclusion in this literature review.

## Pathophysiology of the intersectionality of pain and mental health

Pain is an anticipated side effect following any injury that generally resolves with time, depending on the severity and location of the injury. In some cases, however, pain can become chronic if it persists for longer than 3 months, regardless of attempted treatment with medication^8^. Experiencing chronic pain that does not improve with medication can be quite debilitating to one’s quality of life, ultimately affecting one’s personal health and functional capacity. A report by the Center for Disease Control in 2016 detailed that ~20.4% of the US adult population struggle with chronic pain^9^. Prevalent throughout the population to a similar degree is the existence of mental health disorders. According to the 2021 National Survey on Drug Use and Health, an estimated 22.8% of US adults were living with a mental illness in the past year^10^.

Given the widespread incidence and biological mechanisms of chronic pain and mental health disorders, it comes as no surprise that there exists a bidirectional relationship between the two. Research has shown that there are many overlapping brain regions, specifically the medial prefrontal cortex and limbic system, involved in pain pathways and mood disorders. Patients experiencing pain show greater activity in the medial prefrontal cortex and limbic system, suggesting a functional connection with affect regulation^11^. Thus, the experience of pain and mental health disorders are not activated independently but rather influence each other. The bidirectional relationship is apparent when examining patients with depression. Depressive symptoms are high in patients experiencing pain and pain-free individuals with depression are at a four times increased risk of developing pain^12^. Aside from the overlapping brain regions involved in pain and depression, neurotransmitters have been implicated as major drivers of the association between pain and depression. The neurotransmitter pathways of serotonin and norepinephrine originate in the brainstem nuclei, the same origin as peripheral pathways of pain regulation. Serotonin and norepinephrine projections from the brainstem to the spinal dorsal horn interact with pathways involved in pain control^13^.

Another mental health disorder that highlights this bidirectional relationship is anxiety. Individuals with chronic migraines are twice as likely to be diagnosed with an anxiety disorder, and individuals with anxiety are twice as likely to develop migraines^12^. Chronic pain and mental health disorders exist in a positive feedback loop, each increasing the likelihood of the other. It is crucial to note that the experience of chronic pain can be so crippling that suicide ideation is higher in individuals with chronic pain^11^. Given the unique relationship between chronic pain and mental health disorders, it is important to consider treatment using a multidisciplinary, biopsychosocial approach.

## Mental health as a risk factor for adverse orthopaedic trauma outcomes

Patient**-**reported outcomes and satisfaction scores are vital tools in assessing improvement in quality of life. Elements of these measurements seek to quantify mental health status pre- and post-surgical experience. Several validated scales have been developed to screen patients such as the Patient Health Questionnaire-9 (PHQ-9), Generalized Anxiety Disorder Screener-7 (GAD-7), and the Medical Outcomes Study 36-item Short Form (SF-36)^14–16^. The PHQ-9 screens for depression with a cutoff score of 10 for diagnostic purposes though a score of 5–9 indicates mild depression^14^. The GAD-7 screens for anxiety with a score of 10 or greater indicating diagnosis and a score of 15 or greater signalling severe anxiety^15^. The SF-36 is comprised of both a physical component summary and a mental component summary. The mental component summary measures general mental health status and energy/fatigue^16,17^. Psychological distress is highly prevalent among orthopaedic trauma injury patients screened using the SF-36^18^. Patients with symptoms of depression have been shown to self-report lower function and satisfaction scores as well as higher pain scores at both preoperative and 2-year follow-up after orthopaedic surgical procedures such as hip arthroscopy^19^. In total knee replacement therapies, depression, and anxiety self-reported variables were associated with worse knee outcome scores at 1-year follow-up^20^. Furthermore, poor preoperative SF-36 and General Health Perception scores were associated with longer lengths of hospital stays following hip fracture surgery even when controlling for factors such as age, fracture type, or American Society of Anaesthesiology scores^21^.

### Surgical complications

Post-surgical complications include any deviations from standard postoperative procedures such as infection, readmission, nerve injury, or thromboembolic events. In general surgery elective procedures, readmission rates, and emergency room visits were higher among patients with depression, anxiety, post-traumatic stress disorder, and substance abuse^22^. A systematic review of psychosocial factors and orthopaedic outcomes following rotator cuff tears reported a strong predictive value between mood factors and surgical outcomes^23^.

Compliance with postoperative standards of care helps to reduce complications and improves the recovery process. The use of continued care in a geriatric population of patients undergoing hip fracture surgery led to increased compliance with functional exercises, improved physical outcomes scores, significant reductions in anxiety and depression scores, and overall reduced rates of complications such as lung infections, deep vein thrombosis, or joint stiffness^24^. Aspects of continued care may be applied in other cases of geriatric orthopaedic care to achieve similar outcomes.

There is a marked increase in the risk of postoperative infection in those who suffer from psychiatric diseases^5^. PearlDiver data analysis showed an increased risk of infection following total wrist arthroplasty and arthrodesis was linked to depression and other risk factors like diabetes, age, and post-traumatic arthritis^25^. The pathophysiology of serotonin involved in major depressive disorder has been linked to influence the immune system and trigger inflammation^26,27^.

Pre-existing psychiatric conditions such as depression can negatively impact patient**-**reported outcomes including pain scores. This represents an area of opportunity for orthopaedic physicians by preoperatively identifying mental health status, patient expectations, and postoperative planning can be appropriately tailored to best suit a patient’s individual needs.

### Impact of orthopaedic trauma procedures on mental health

The adverse effects of orthopaedic injuries on mental health have been well established. There is considerable research suggesting injured athletes experience increased risks of depression and anxiety, while less has been explored regarding orthopaedic trauma injuries^28^. Orthopaedic trauma injuries can be quite severe, often drastically altering one’s physical capabilities, which can be core to one’s identity. Survivors of orthopaedic trauma injuries experience lingering issues that interfere with their quality of life and often struggle with post-traumatic stress disorder, anxiety, and depression^29^. This kind of psychological distress has been established as a strong predictor of adverse postoperative outcomes^30^. A key component in adverse mental health outcomes following orthopaedic trauma is chronic pain, which is a frequent side effect of orthopaedic trauma surgeries^31^. A systematic review exploring the pain and psychological outcomes following traumatic musculoskeletal injuries found that pain persists up to 84 months following the traumatic injury^32^.

A unique injury that distinguishes orthopaedic trauma from most other orthopaedic specialties is traumatic limb amputation. Limb amputations of a traumatic aetiology have a distinct impact compared to anticipated amputations from existing medical conditions. Unexpected amputation is a devastating event that drastically alters the course of one’s life. Routine follow-up care and regaining functional capacity encompass life post-amputation. Amputees of traumatic injury can experience psychological trauma with extended periods of grieving and severe mental health complications from the sudden change in their quality of life^33^. There is a critical need to focus on mental health outcomes following orthopaedic trauma to optimize surgical outcomes and patient quality of life.

### Opioid use following orthopaedic trauma

When considering the effect of depression on surgical outcomes, a special consideration would be the use of opioid analgesia. Pain management in modern medicine has a variety of options at the physician’s disposal, but opioid**-**based medication is commonly used for many postoperative patients due to its inexpensive, yet powerful effect. In fact, it is estimated that up to 80% of patients receive opioid medication after low-risk surgery, with the most common formulas including oxycodone and hydrocodone^34^. Orthopaedic surgery is no different as orthopaedists are the third highest prescribers of opioid medications among any US medical specialty, and were roughly seven times more likely to prescribe opioids than general practitioners for noncancer chronic pain^35,36^. Thus, it would be reasonable to conclude that opioids have positioned themselves as a mainstay in pain management for the orthopaedic trauma patient. However, their use is not benign. Opioid medications administered at prescribed doses have the potential to place the opioid-naive patient at risk for tolerance and dependence^37^. In addition, 14.7% of patients at an orthopaedic clinic reported use of opioid medication longer than, and sometimes at higher doses, than their original prescriptions^38^. Should the patient have a preoperative diagnosis of depression and be using prescription antidepressants, their risk for chronic opioid use in the postoperative period is only worsened^39^. In some instances, of patients who were interviewed 1–2 months after their injury, roughly 28% reported continued opioid use despite being well beyond a recovery period that necessitates opioid analgesia^40^. Even if a patient is to be well-controlled in the postoperative period, roughly 25% of some orthopaedic trauma patient populations exhibit risk factors for opioid abuse and dependency^41^. Be it the opioid itself, or surgery being an independent risk factor for chronic opioid use, the orthopaedic trauma patient is at risk of dependency and abuse at some stage of their healthcare experience^42^.

The issue is only magnified by the fact that opioid abuse and worsening symptoms of depression exist in a bidirectional relationship. As the duration of use of opioid medications increases, the worsening of pre-existing depression also increases. In the inverse, patients with pre-existing depression are known to have higher rates of opioid abuse, thus creating a potential cycle of worsening depression and dependency on opioids^43^. Even when patients are not abusing opioids, patients who are prescribed them for durations of longer than 30 days are at increased risk of new**-**onset depression^44^. Should patients require use for greater than 72 days, they are at a 40% increased risk for a new depressive episode compared to those who required use for less than 45 days^45^.

Knowing the prevalent use of opioids by orthopaedic surgery and the associations of opioids and risk for new or worsening depression, it seems even postoperative pain management may compound the adverse effect depression has on surgical outcomes. Should the dependency on opioid analgesia, as monotherapy or the dominant medication in a multimodal strategy, be decreased in favour of non-opioid techniques, it is possible that post-surgical outcomes could be improved by limiting the impact of opioid**-**exacerbated depression.

## Conclusion

In conclusion, mental health disorders, particularly anxiety and depression, have a significant impact on orthopaedic trauma surgery outcomes. Patients with orthopaedic trauma injuries have a high incidence of psychiatric disorders, and negative mental health outcomes increase the risk of morbidity and mortality. Chronic pain and mental health disorders have a bidirectional relationship, influencing each other through overlapping brain regions and neurotransmitter pathways. Preoperative and postoperative mental health interventions, including screening with validated scales, can improve patient outcomes and satisfaction scores. Orthopaedic surgeons and other healthcare professionals caring for orthopaedic trauma patients should consider multidisciplinary, biopsychosocial treatment approaches that address both physical and mental health. Further research in this area is necessary to develop effective interventions that improve mental health outcomes and overall surgical outcomes in orthopaedic trauma patients.

## Ethical approval

Ethics approval was not required for this review.

## Consent

Informed consent was not required for this review.

## Source of funding

None.

## Author contribution

J.W.: study concept, design, writing the paper, editing, submission. A.V.: writing the paper. N.S.: writing the paper. C.S.: writing the paper. B.G.: supervision, editing. D.C.: supervision, editing. G.H.: supervision, editing.

## Conflicts of interest disclosure

There are no conflicts of interest.

## Research registration unique identifying number (UIN)

None.

## Guarantor

Jonathan Weinerman.

## Data availability statement

None.

## Provenance and peer review

None.
